# AQP9 and ZAP70 as immune-related prognostic biomarkers suppress proliferation, migration and invasion of laryngeal cancer cells

**DOI:** 10.1186/s12885-022-09458-8

**Published:** 2022-04-28

**Authors:** Li Ren, Ping Li, Zhouping Li, Quan Chen

**Affiliations:** 1 Department of Biochemistry, Jinzhou Medical University, Jinzhou, 121000 Liaoning China; 2 Department of Oncology, The First Affiliated Hospital of Jinzhou Medical University, Jinzhou, 121000 Liaoning China; 3 Department of Surgery, The First Affiliated Hospital of Jinzhou Medical University, Jinzhou, 121000 Liaoning China; 4 Department of Anesthesiology, The First Affiliated Hospital of Jinzhou Medical University, No.2, Section 5, Renmin Street, Guta District, Jinzhou, 121000 Liaoning China

**Keywords:** Laryngeal cancer, Immune-related genes, Prognosis, AQP9, ZAP70, Immune microenvironment, Progression

## Abstract

**Background:**

Laryngeal cancer represents a common malignancy that originates from the larynx, with unfavorable prognosis. Herein, this study systematically analyzed the immune signatures of laryngeal cancer and to evaluate their roles on tumor progression.

**Methods:**

Differentially expressed immune-related genes (IRGs) were screened between laryngeal cancer and normal tissues from TCGA dataset. Then, two prognosis-related IRGs AQP9 and ZAP70 were analyzed by a series of survival analysis. Based on them, molecular subtypes were constructed by unsupervised cluster analysis. Differences in survival outcomes, HLA expression and immune cell infiltrations were assessed between subtypes. Expression of AQP9 and ZAP70 was validated in laryngeal cancer tissues and cells by RT-qPCR and immunohistochemistry. After silencing and overexpressing AQP9 and ZAP70, CCK-8, EdU, wound healing and transwell assays were performed in TU212 and LCC cells.

**Results:**

Totally, 315 IRGs were abnormally expressed in laryngeal cancer. Among them, AQP9 and ZAP70 were distinctly correlated to patients’ prognosis. Two subtypes were developed with distinct survival outcomes, HLA expression and immune microenvironment. Low expression of AQP9 and ZAP70 was confirmed in laryngeal cancer. AQP9 and ZAP70 up-regulation distinctly suppressed proliferation, migration, and invasion of laryngeal cancer cells. The opposite results were investigated when their knockdown.

**Conclusions:**

Our findings revealed the roles of AQP9 and ZAP70 in progression of laryngeal cancer, and suggested that AQP9 and ZAP70 could potentially act as candidate immunotherapeutic targets for laryngeal cancer.

**Supplementary Information:**

The online version contains supplementary material available at 10.1186/s12885-022-09458-8.

## Background

Laryngeal cancer represents a commonly diagnosed tumor in respiratory tract, accounting for around 2.4% of systemic malignancies globally each year [1]. Incidence and prevalence have separately raised by 12 and 24% over the past 3 decades [2]. Laryngeal cancer patients experience badly impaired vocal, respiratory, and swallowing functions with unfavorable prognoses [3]. The onset of laryngeal cancer is dormant and most of patients (~ 60%) present with advanced (stage III and IV) disease when diagnosed [4]. Tendency to local invasion and cervical lymph node metastases badly affect patients’ survival outcomes [5]. Surgical resection is still the main therapeutic approach for laryngeal cancer though the treatment strategies have undergone considerable progress in recent years [6]. As an example, radiotherapy technique like stereotactic radiation therapy and particle beam radiation therapy offers advantages regarding radiation precision [7]. Nevertheless, efficacy of advanced therapies is still unsatisfactory and its five-year survival rate has declined from 66 to 63% in the recent years due to indistinct mechanisms of initiation and development [8]. Hence, it is of importance to reveal the pathogenesis of laryngeal cancer, ascertain diagnostic signatures as well as probe efficient novel therapeutic targets [9].

Immunotherapy has dramatically altered the therapy landscape for laryngeal cancer patients [1]. Nevertheless, scarce integrated profiling of laryngeal cancer immune microenvironment may hamper the clinical application of immunotherapy and limit eligible patients [10]. Emerging evidence demonstrates that tumor microenvironment exerts a key role in cancer development and progression [11, 12]. Dysregulation of immune system may become a main cause of cancer progression and affects the efficacy of immunotherapy [13]. Hence, accurate regulation of immune-related gene (IRG) expression is of importance to generate a robust immunity [14, 15]. Herein, this study systematically analyzed the immunogenomic landscape in laryngeal cancer and screened two prognosis-related IRGs aquaporin 9 (AQP9) and zeta chain of T cell receptor associated protein kinase 70 (ZAP70) that exhibited well predictive performance for laryngeal cancer patients’ prognosis after validation. AQP9 protein is a family member of water-selective membrane channels, which allows passage of a broad range of noncharged solutes and can stimulate urea transport and osmotic water permeability [16]. Moreover, this protein is involved in mediating specialized leukocyte functions such as immunological response and bactericidal activity. ZAP70 gene encodes an enzyme that belongs to the protein tyrosine kinase family, which exerts an important function in T-cell development and lymphocyte activation [17]. Based on the two signatures, we established two molecular subtypes, which reflected the immune microenvironment. Also, their up-regulation inhibited proliferation, migration, and invasion of laryngeal cancer cells. Hence, AQP9 and ZAP70 might become candidate therapeutic targets and robust prognostic signatures for laryngeal cancer.

## Materials and methods

### Data collection and preprocessing

High-throughput sequencing (HTSeq) counts and Fragments Per Kilobase of transcript per Million mapped reads (FPKM) and clinical information of laryngeal cancer patients were retrieved from the Cancer Genome Atlas database (TCGA; http://cancergenome.nih.gov/). Totally, 111 laryngeal cancer and 12 normal control specimens were included in this study. Clinical features of laryngeal cancer patients were listed in Supplementary Table 1. A total of 1509 immune-related genes (IRGs) were downloaded from the ImmPort database (https://immport.niaid.nih.gov). The reference human genome annotation file v22 was obtained from the GENCODE database (https://www.gencodegenes.org). ENSG number was transformed to gene symbol. Then, the genes whose expression value was all 0 were removed and the expression profile of IRGs was extracted.

### Differential expression analysis

The mRNA expression of IRGs between laryngeal cancer and control samples was compared by DESeq2 [18] and edgeR packages [19]. Genes whose median and variance of counts ≤20% of the median and variance of counts of all genes were filtered. IRGs with adjusted *p* < 0.05 and |log2fold-change| > 1 were screened, which were visualized into volcano plots. Differentially expressed IRGs were identified by intersection of DESeq2 and edgeR analyses.

### Screening prognosis-related IRGs

Laryngeal cancer samples were randomly split into training set and testing set according to 2:1. After normal distribution test, there was no significant difference in clinical information (age, gender and clinical stage) between training set and testing set by student’ t test or wilcox test. In the training set, univariate cox regression analysis was used to preliminary screen differentially expressed IRGs. IRGs with *p* < 0.05 were included for clinical factor correction and multivariate cox regression analysis, with repeated 1000 times. Finally, IRGs that were repeated more than 200 times were considered as candidate prognostic signatures. Using the surv_cutpoint function in the survminer package, the cutoff values of candidate prognostic signatures were determined, and samples were divided into high and low expression groups in training and test sets. The log-rank method was used to assess the difference in survival between groups.

### Unsupervised cluster analysis

ConsensusClusterPlus package was employed for unsupervised cluster analysis of laryngeal cancer samples with complete clinical information [20]. The consensus matrix was conducted according to k values. Empirical cumulative distribution function (CDF) diagrams were applied for revealing the consensus distribution for each k and delta area plots and item tracking plots was depicted to determine the stability of clustering. Kaplan-Meier curves of overall survival were depicted between subtypes and were evaluated with log-rank test. Clinical features were compared between subtypes with Fisher test. Furthermore, five-year survival rate was compared between subtypes. Differentially expressed IRGs between subtypes were screened by DESeq2 and edgeR packages.

### Functional enrichment analysis

Gene Ontology (GO) and Kyoto Encyclopedia of Genes and Genomes (KEGG) enrichment analyses of differentially expressed IRGs were performed by clusterProfiler package [21]. Terms with adjusted *p* < 0.05 were significantly enriched.

### Assessment of HLA expression and immune cell infiltration

HLA-A, HLA-B and HLA-C expression was compared between subtypes with wilcox test. The ratio of the content of 22 immune cells in each sample was inferred by CIBERSORT algorithm [22].

### Patients and specimens

Totally, 30 pairs of primary human laryngeal cancer tissues and adjacent normal tissues were collected in the First Affiliated Hospital of Jinzhou Medical University (Jinzhou, China), under the guidance of a skilled pathologist. This research was approved by the Ethics Committee of the First Affiliated Hospital of Jinzhou Medical University (2020008), and the research protocol was implemented under its supervision. All patients provided written informed consent. After surgical resection, tissue specimens were immediately frozen in liquid nitrogen and stored at − 80 °C.

### Real-time quantitative polymerase-chain reaction (RT-qPCR)

Tissues or cells were lysed by Trizol. After centrifugation of 12000 rpm at 4 °C for 5 min, the supernatant was collected. Then, chloroform was added and stood on the ice, followed by centrifugation at 12000 rpm for 15 min at 4 °C. The upper water phase was then collected, which was added by isopropanol. Following centrifugation, total RNA was extracted. UV spectrophotometer was used to measure the concentration of RNA and the absorbance at 260/280 nm. Using PrimeScript™ RT reagent Kit (TakaRa, China), RNA was reverse transcribed into cDNA. Primers of GAPDH, AQP9 and ZAP70 were synthesized by Shenyang Wanze Biological Technology Co., Ltd. (China), as follows: GAPDH: 5′-GGACCTGACCTGCCGTCTAG-3′ (forward), 5′-TAGCCCAGGATGCCCTTGAG-3′ (reverse); AQP9: 5′-GAAGAGCAGCTTAGCGAAAGA-3′ (forward), 5′-ACAGCCACATCCAAGGACAAT-3′ (reverse); ZAP70: 5′- CGAGCGTGTATGAGAGCCC-3′ (forward), 5′-ATGAGGAGGTTATCGCGCTTC-3′ (reverse). RT-qPCR was performed on ABI7500 PCR instrument (ABI, USA) and PCR kit (TakaRa, China). Relative expression was calculated with 2^-ΔΔCT^ method.

### Immunohistochemistry

Tissue or cell sections were dewaxed, re-hydrated, retrieved by antigen and blocked. Thereafter, the sections were incubated with primary antibodies against AQP9 (1:200; NBP1-30865; Novus, USA), and ZAP70 (1:100; ab32429; Abcam, USA) at 4 °C overnight. Afterwards, membrane was incubated with HRP-conjugated secondary antibodies (1:200; SA00001-2; Proteintech, USA) at room temperature for 15 min. Thereafter, the sections were stained with DAB and hematoxylin, and were dehydrated and mounted with coverslips. According to H-score method, the expression of AQP9 and ZAP70 was quantified.

### Cell culture

Human laryngeal cancer cells TU212 and LCC as well as human immortalized keratinocyte cells HaCaT were purchased from the Shanghai Cell Bank of the Chinese Academy of Sciences (Shanghai, China). TU212 and LCC cells were cultured in RPMI 1640 (Thermo Scientific, USA) and 20% fetal bovine serum (FBS; Hyclone, MA, USA). HaCaT cells were cultured with MEM (Thermo Scientific, USA) and 15% FBS. All cells were cultured in a 5% CO_2_ incubator at 37 °C.

### Cell transfection

TU212 and LCC cells were plated in a 6-well plate (3.0 × 10^5^ cells/well). SiRNA against AQP9 (si-AQP9; Ribo, Suzhou, China; sequences: siRNA#1: 5′-GAGGCCTCATCTATGTTCT-3′; siRNA#2: 5′-CAGTCGAGGACGTTTTGGA-3′; siRNA#3: 5′-CATACCCAGCTCCGTATCT-3′) and ZAP70 (si-ZAP70; Ribo, Suzhou, China; sequences: siRNA#1: 5′-GCAACGTCCTGCTGGTTAA-3′; siRNA#2: 5′-GCACCCGAATGCATCAACT-3′; siRNA#3: 5′-GCGCGATAACCTCCTCATA-3′), siRNA negative control (si-NC; Ribo, Suzhou, China), AQP9 and ZAP70 overexpression plasmids (Hanbio, Shanghai, China) and empty vector were transfected into cells via X-tremeGENE siRNA Transfection Reagent (Roche, Shanghai, China) or X-tremeGENE HP DNA Transfection Reagent (Roche, Shanghai, China). After transfection for 48 h, RT-qPCR was used to examine AQP9 and ZAP70 expression.

### Western blot

Total protein was extracted from TU212 and LCC cells by RIPA lysis. Extracted protein was separated by SDS-PAGE gel and transferred onto PVDF membrane (Millipore, USA). Then, membrane was blocked by 3% BSA for 30 min and incubated with primary antibodies against AQP9 (1:1000; NBP1-30865; Novus, USA), ZAP70 (1:500; ab32429; Abcam, USA) and GAPDH (1:1000; 10,494-1-ap; Proteintech, USA) at 4 °C overnight. Afterwards, membrane was incubated with secondary antibodies (1:5000; SA00001-2; Proteintech, USA) at room temperature for 1 h. Protein bands were visualized by ECL kit (Thermo Scientific, USA).

### Cell counting kit-8 (CCK-8)

Transfected TU212 and LCC cells were seeded onto a 96-well plate (3.0 × 10^3^ cells/well). After 4 h, mixture of CCK-8 (Dojindo, Japan) and serum-free culture medium (1:10) was added to each well and incubated for 1 h. Automatic microplate reader was used to measure absorbance value at 450 nm wavelength at 0, 24, 48, 72, 96 h.

### 5-Ethynyl-2′-deoxyuridine (EdU) staining

kFluor555-EdU cell proliferation detection kit (KGA337-500; KeyGEN BioTECH, Jiangsu, China) was used to detect cell proliferation. Transfected TU212 and LCC cells were plated onto a 12-well plate (2.0 × 10^4^ cells/well). After 24 h, cells were incubated with 20 μM 1 × EdU. Then, cells were fixed by 4% paraformaldehyde (1 ml/well; G1113; Servicebio, Wuhan, China). Cells were incubated with 1 ml 0.5% Triton X-100 in PBS for 20 min at room temperature. 500 μl Click-iT reaction mixture were added to each well and incubated for 30 min in the dark. Then, cells were incubated with 5 μg/mL 1 × Hoechst33342 (500 μl/well) for 30 min in the dark. Under an inverted fluorescence microscope (Nikon Eclipse; Nikon, Japan), images were acquired. EdU was investigated with 555 nm excitation light, and Hoechst was investigated with 350 nm excitation light.

### Wound healing

Using a marker pen, a horizontal line was drawn on the back of the 6-well plate. Transfected TU212 and LCC cells were inoculated into the 6-well plate, and cells covered the bottom of the plate after being attached to the wall. A 200 μl pipette tip was used to make cell scratches perpendicular to the well plate. The cell culture medium was aspirated and the well plate was rinsed for three times with PBS to wash away the cell debris generated by the scratch. Then, serum-free medium was added. After 0, 12 and 36 h, images were acquired and crystal violet was dissolved with 30% acetic acid. The absorbance value at 570 nm wavelength was detected.

### Transwell

For invasion assay, Matrigel was spread on the upper chamber of the transwell chamber, without Matrigel for migration assay. 600 μl 10% FBS medium was added to the lower chamber. Transfected TU212 and LCC cells were seeded onto transwell chamber (2.0 × 10^4^ cells/well; Corning, Germany). Then, cells were cultured in a 37 °C and 5% CO_2_ incubator. After 24 h, cells were fixed by 4% paraformaldehyde (1 ml/well; G1113; Servicebio, Wuhan, China) for 30 min. Cells were stained with 0.1% crystal violet for 15 min. Images were acquired under an optical microscope (Canon, China) and absorbance value at 570 nm wavelength was detected.

### Statistical analysis

Receiver operator characteristic (ROC) curves and area under the curve (AUCs) were conducted to assess the diagnostic value of AQP9 and ZAP70 expression in laryngeal cancer. Each experiment was repeated at least three times. Data were expressed as mean ± standard deviation. Comparisons between groups were performed by student’ t test, one-way or two-way analysis of variance. *P* < 0.05 was statistically significant.

## Results

### Identification of prognosis-related IRGs in laryngeal cancer

**Fig. 1:**
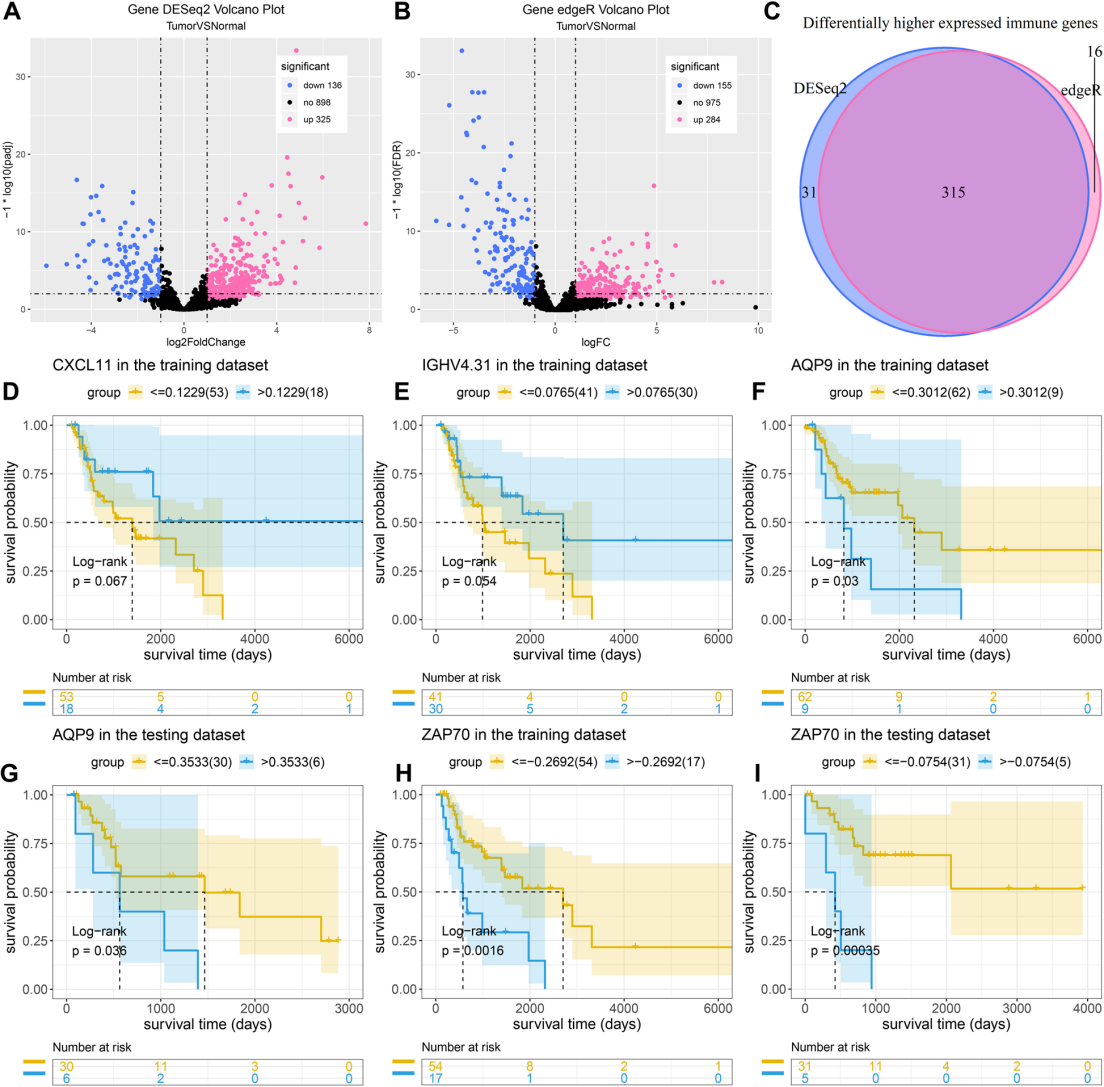
Identification of prognosis-related IRGs in laryngeal cancer. **A**, **B** Volcano plots of up- (red) and down-regulated (blue) IRGs in laryngeal cancer than control tissues by DESeq2 and edgeR. **C** Venn diagram of common differentially expressed IRGs by intersection of DESeq2 and edgeR. **D**, **E** Kaplan-Meier curves of CXCL11 and IGHV4.31 in the training set. **F**, **G** Kaplan-Meier curves of AQP9 in the training and validation sets. **H**, **I** Kaplan-Meier curves of ZAP70 in the training and validation sets. *P* values were determined by log-rank tests

In this study, 111 laryngeal cancer and 12 adjacent control tissues were obtained from TCGA dataset. With the screening criteria of adjusted *p* < 0.05 and |log2fold-change| > 1, we identified 325 up- and 136 down-regulated IRGs in laryngeal cancer by DESeq2 (Fig. 1A; Supplementary Table 2). Meanwhile, 284 up- and 155 down-regulated IRGs were screened in laryngeal cancer by edgeR (Fig. 1B; Supplementary Table 3). After intersection, 315 differentially expressed IRGs were identified in this study (Fig. 1C). Prognosis-related IRGs were then explored by univariate cox regression analysis and correction of clinical factors, AQP9, CXCL11, IGHV4.31, PDGFA and ZAP70 were significantly correlated to laryngeal cancer prognosis (Table 1). To further validate the prognostic value of these genes, we divided laryngeal cancer samples into training and validation sets. Our results showed that patients with high expression of CXCL11 and IGHV4.31 indicated prolonged survival time than those with their low expression in the training set (Fig. 1D, E). However, there was no statistical significance. Furthermore, high expression of AQP9 and ZAP70 was significantly correlated to favorable survival outcomes compared to their low expression in laryngeal cancer (Fig. 1F-I). Therefore, AQP9 and ZAP70 were identified as prognostic IRGs of laryngeal cancer.

**Table 1:** Screening prognosis-related IRGs in laryngeal cancer

	Univariate cox regression analysis			Correction of clinical factors		
Factors	Coef	HR	*P*-value	Coef	HR	*P*-value
AQP9	0.0300	1.0304	0.0112	0.0305	1.0310	0.0089
CXCL11	0.0043	1.0043	0.0362	0.0057	1.0057	0.0083
IGHV4.31	0.0011	1.0011	0.0086	0.0013	1.0013	0.0065
PDGFA	0.0150	1.0150	0.0484	0.0197	1.0199	0.0143
ZAP70	−0.1296	0.8784	0.0208	−0.1270	0.8808	0.0234

### Construction of two molecular subtypes with distinct survival outcomes and biological functions based on AQP9 and ZAP70 expression profiles

**Fig. 2:**
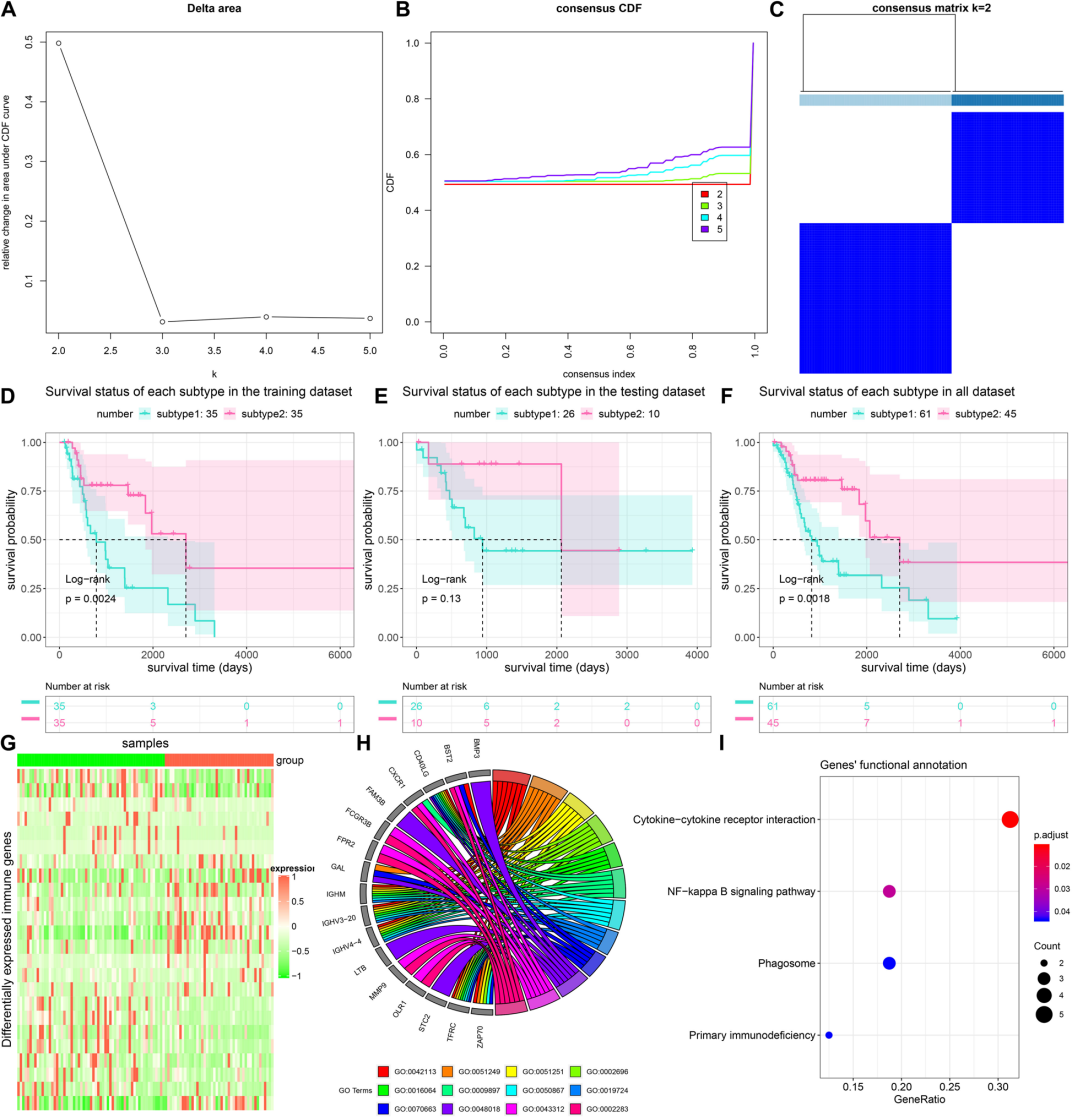
Characterization of two molecular subtypes with distinct survival outcomes and biological functions for laryngeal cancer. **A**-**C** Identification of two molecular subtypes of laryngeal cancer by ConsensusClusterPlus package. **D**-**F** Survival analysis of two subtypes in training set, testing set and entire set. *P* values were determined using log-rank test. **G** Heatmap of expression patterns of 24 IRGs in two subtypes. **H** GO enrichment results of 24 IRGs. **I** KEGG pathway enrichment of 24 IRGs

By unsupervised cluster analysis, we constructed two molecular subtypes of laryngeal cancer using ConsensusClusterPlus package based on AQP9 and ZAP70 expression profiles (Fig. 2A-C). Survival differences between subtypes were further analyzed. Our data showed that patients in subtype 2 exhibited prolonged survival time than those in subtype 1 in the training set (Fig. 2D), testing set (Fig. 2E) and entire set (Fig. 2F). Table 2 listed the clinical features between subtypes. We found that there was significant difference in survival status between subtypes. To uncover the underlying molecular mechanisms, the expression of IRGs between subtypes was compared by DESeq2 and edgeR. After intersection, 24 IRGs (GAL, TFRC, MMP9, CD40LG, AQP9, NR2E1, STC2, ZAP70, IL1R2, BST2, ACTA1, BMP3, FCGR3B, CXCR1, FPR2, RXFP1, OLR1, DES, FAM3B, IGLV10-54, IGHM, IGHV3-20, LTB, and IGHV4-4) exhibited differential expression between subtypes (Fig. 2G). GO enrichment analysis revealed that 24 IRGs were significantly associated with B cell activation, regulation of lymphocyte activation, positive regulation of lymphocyte activation, positive regulation of leukocyte activation, immunoglobulin mediated immune response, external side of plasma membrane, positive regulation of cell activation, B cell mediated immunity, regulation of leukocyte proliferation, receptor ligand activity, neutrophil degranulation and neutrophil activation involved in immune response (Fig. 2H). Furthermore, cytokine-cytokine receptor interaction, NF-kappa B signaling pathway, phagosome and primary immunodeficiency pathways were significantly related to these IRGs (Fig. 2I). Above data indicated that these IRGs mainly exerted immune-related functions during laryngeal cancer progression.

**Table 2:** Clinical features of two molecular subtypes

Features	Subtype 1	Subtype 2	*P*-value
Age			
Age ≤ 60	32	15	0.0746
Age > 60	29	30	
Gender			
Male	49	37	1
Female	12	8	
Stage			
Stage I	2	1	0.9221
Stage II	7	4	
Stage III	16	10	
Stage IV	36	30	
Survival status			
Dead	34	14	0.0175
Alive	27	31	

### Two molecular subtypes with distinct immune microenvironment

**Fig. 3:**
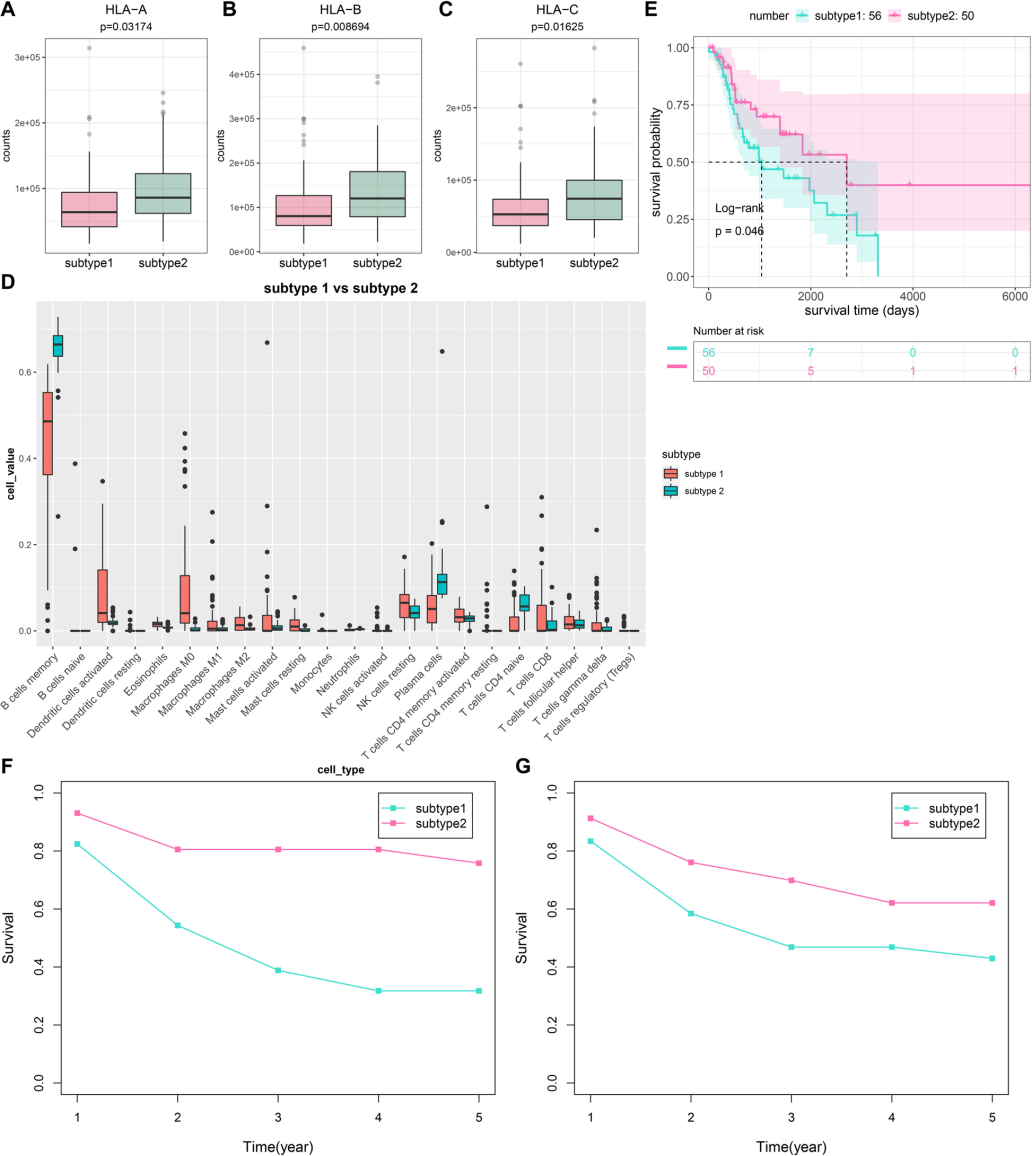
Two molecular subtypes with distinct immune microenvironment in laryngeal cancer. **A**-**C** Box plots of HLA-A, HLA-B and HLA-C expression in subtype 1 and subtype 2. **D** Box plots of infiltration levels of immune cells in two subtypes. **E** Survival probability of two subtypes based on immune cells. *P* value was calculated with log-rank test. **F** Assessment of five-year survival rate between subtypes based on AQP9 and ZAP70 expression. **G** Five-year survival rate between subtypes based on immune cells

We found that subtype 2 had increased expression of HLA-A, HLA-B and HLA-C than subtype 1 (Fig. 3A-C). Higher infiltration levels of B cells memory, plasma cells and T cells CD4 naïve were found in subtype 2 compared to subtype 1 (Fig. 3D). Meanwhile, there were increased infiltration levels of dendritic cells activated, macrophages M0, macrophages M1, macrophages M2, mast cells activated, mast cells resting, NK cells resting, T cells CD4 memory activated, T cells CD8, T cells follicular helper and T cells gamma delta in subtype1 compared to subtype 2. These data indicated the distinct immune microenvironment between subtypes. Based on immune cells, laryngeal cancer samples were divided into subtype 1 and 2. Prolonged survival time was found in subtype 2 (Fig. 3E). Five-year survival rate was also compared between subtypes. In Fig. 3F, more favorable survival outcomes were observed in subtype 1 than subtype 2 based on AQP9 and ZAP70 expression. The consistent results were found based on immune cells (Fig. 3G).

### Validation of AQP9 and ZAP70 expression in laryngeal cancer

**Fig. 4:**
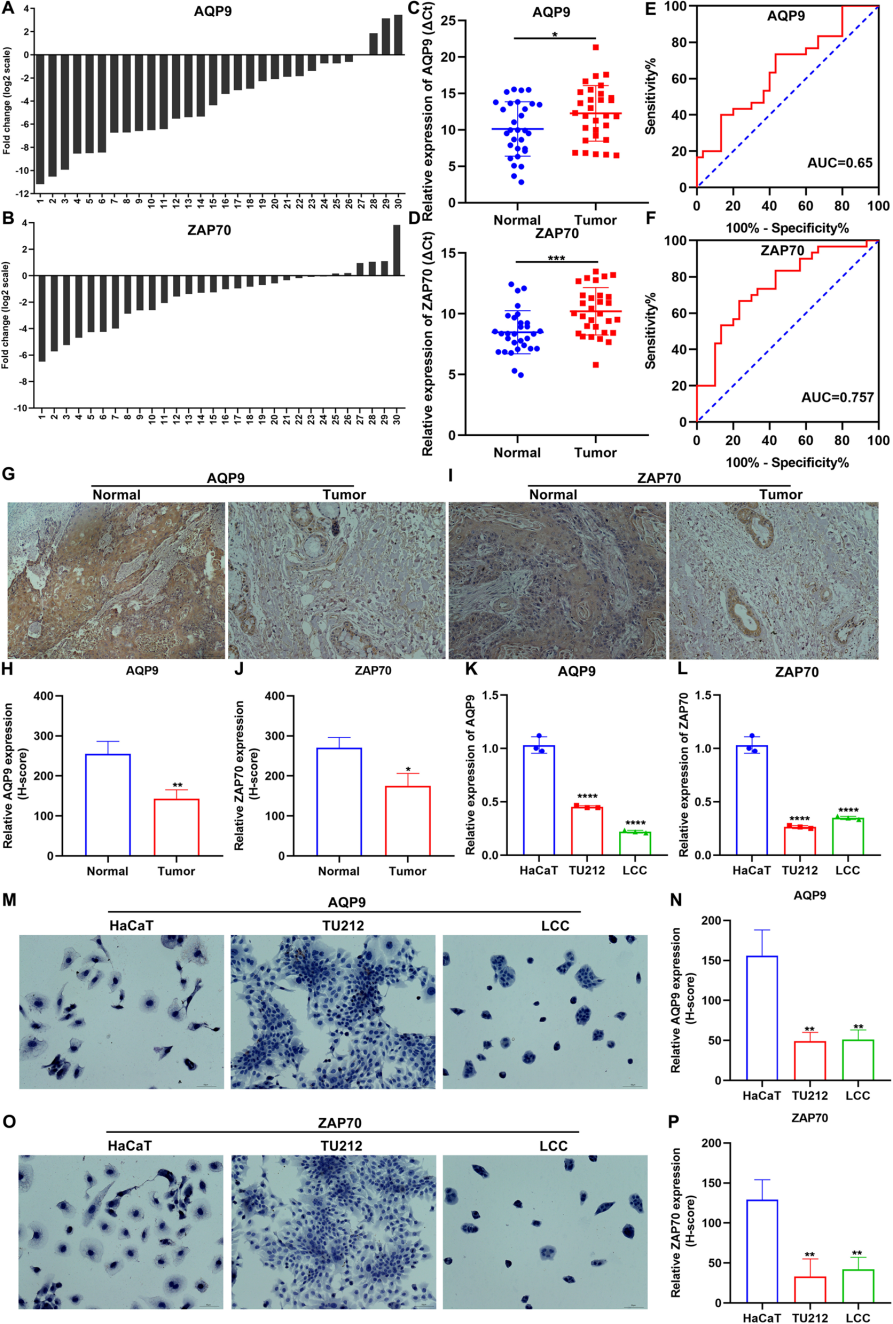
Validation of AQP9 and ZAP70 expression in laryngeal cancer. **A**, **B** RT-qPCR for expression of AQP9 and ZAP70 in each laryngeal cancer sample. **C**, **D** RT-qPCR for expression of AQP9 and ZAP70 in laryngeal cancer and normal tissues. **E**, **F** ROC curves of AQP9 and ZAP70 expression for assessing the diagnostic value of their expression. **G**-**J** Immunohistochemistry analysis of expression of AQP9 and ZAP70 in laryngeal cancer and normal tissues. Scale bar, 50 μm. **K**, **L** RT-qPCR for expression of AQP9 and ZAP70 in HaCaT, TU212 and LCC cells. **M**-**P** Immunohistochemistry analysis of expression of AQP9 and ZAP70 in HaCaT, TU212 and LCC cells. Scale bar, 50 μm. *P < 0.05; **p < 0.01; ***p < 0.001; ****p < 0.0001

By RT-qPCR, we detected AQP9 and ZAP70 expression in 30 pairs of laryngeal cancer and adjacent control tissues. Figure 4A, B showed the expression distribution of AQP9 and ZAP70 in laryngeal cancer specimens. Most of them had relatively low expression levels of AQP9 and ZAP70. Furthermore, we compared the expression of AQP9 and ZAP70 in tumor and normal tissues. Lower expression of AQP9 and ZAP70 was found in tumor tissues compared to normal tissues (Fig. 4C, D). To explore the clinical significance of the expression of AQP9 and ZAP70 in the diagnosis of laryngeal cancer, ROC curves were drawn to evaluate the diagnostic value of their expression. AUCs of AQP9 and ZAP70 were 0.65 and 0.757, respectively (Fig. 4E, F). Immunohistochemistry results also confirmed that AQP9 and ZAP70 expression was significantly lower in tumor tissues relative to normal tissues (Fig. 4G-J). Also, we detected AQP9 and ZAP70 expression in HaCaT, TU212 and LCC cells by RT-qPCR and immunohistochemistry. Our data showed that lower AQP9 and ZAP70 expression was found in TU212 and LCC cells than HaCaT cells (Fig. 4K-P).

### AQP9 and ZAP70 up-regulation inhibits proliferation of laryngeal cancer cells

**Fig. 5:**
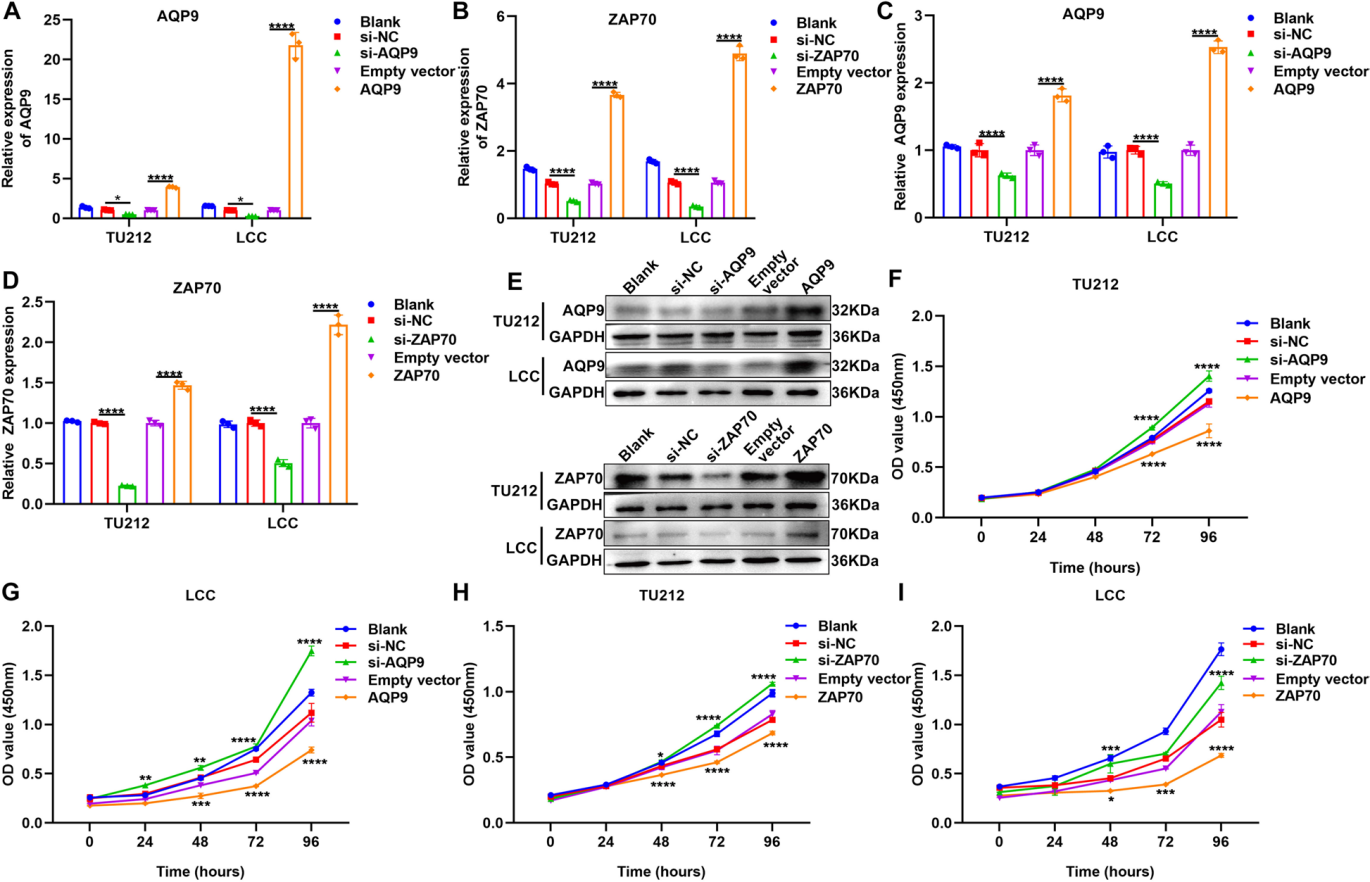
AQP9 and ZAP70 up-regulation inhibits cell viability of laryngeal cancer. **A**, **B** RT-qPCR for AQP9 and ZAP70 mRNA expression in TU212 and LCC cells transfected with siRNAs against AQP9 and ZAP70 or their overexpression plasmids. **C**-**E** Western blot for AQP9 and ZAP70 expression in TU212 and LCC cells transfected with siRNAs against AQP9 and ZAP70 or their overexpression plasmids. **F**, **G** CCK-8 for cell viability of TU212 and LCC cells transfected with si-AQP9 or AQP9 overexpression plasmid. **H**, **I** CCK-8 for cell viability of TU212 and LCC cells transfected with si-ZAP70 or ZAP70 overexpression plasmid. *P < 0.05; **P < 0.01; ***p < 0.001; ****p < 0.0001

**Fig. 6:**
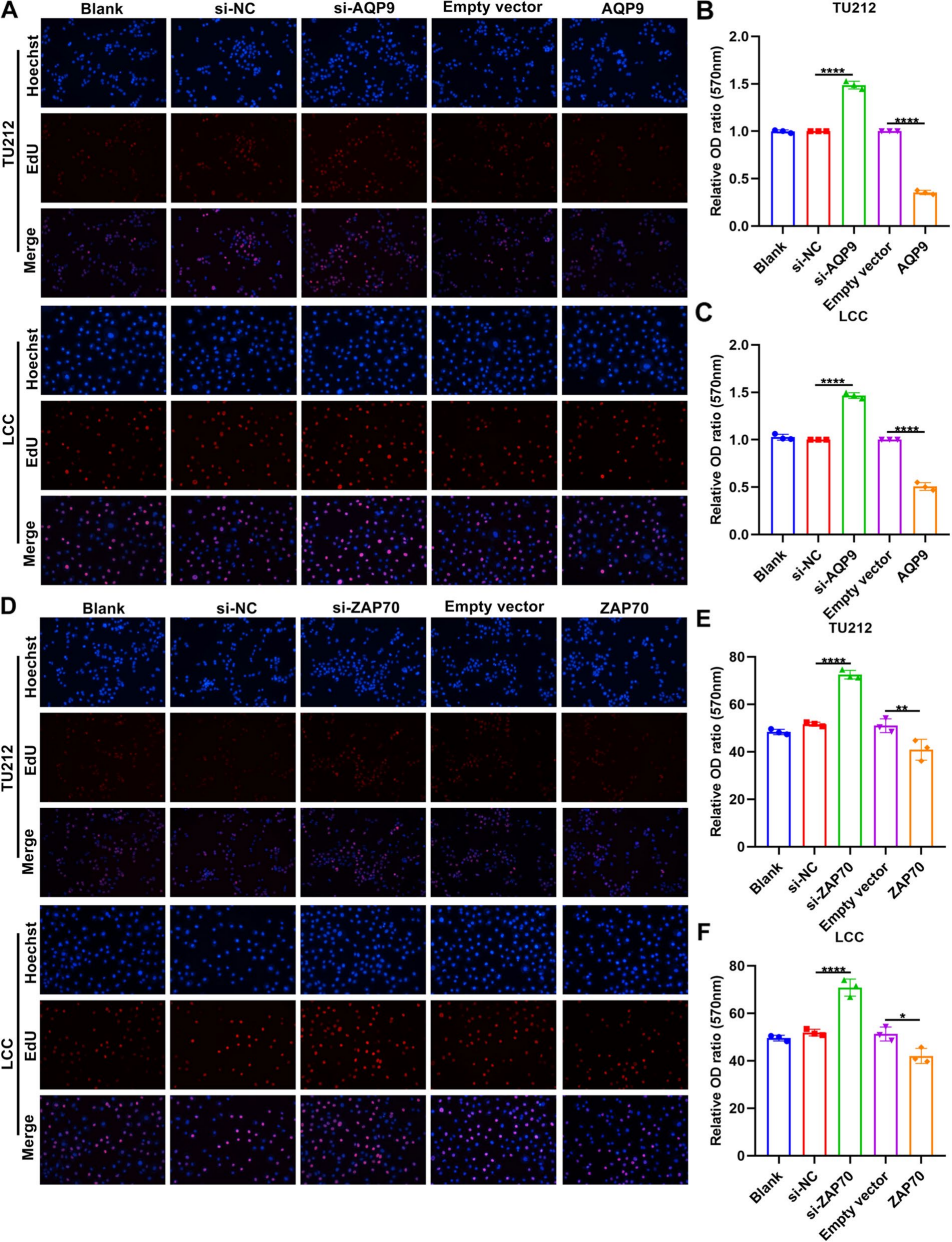
AQP9 and ZAP70 overexpression suppresses proliferation of laryngeal cancer cells. **A**-**C** EdU staining for proliferation of TU212 and LCC cells after transfection with si-AQP9 or AQP9 overexpression plasmid. **D**-**F** EdU staining for proliferation of TU212 and LCC cells after transfection with si-ZAP70 or ZAP70 overexpression plasmid. *P < 0.05; **p < 0.01; ****p < 0.0001

To investigate biological functions of AQP9 and ZAP70 during laryngeal cancer progression, we separately silenced and overexpressed AQP9 and ZAP70 in TU212 and LCC cells. RT-qPCR results demonstrated that AQP9 and ZAP70 mRNA expression was distinctly decreased by their siRNAs and was overexpressed by their overexpression plasmids in laryngeal cancer cells (Fig. 5A, B). Similar results were observed at the protein levels (Fig. 5C-E). CCK-8 was presented to evaluate the cell viability of laryngeal cancer cells. As a result, silencing AQP9 (Fig. 5F, G) and ZAP70 (Fig. 5H, I) distinctly promoted cell viability of TU212 and LCC cells. Oppositely, overexpression of AQP9 (Fig. 5F, G) and ZAP70 (Fig. 5H, I) significantly lowered cell viability of TU212 and LCC cells. EdU staining was utilized for assessing cell proliferation of laryngeal cancer cells. Our data demonstrated that AQP9 knockdown significantly increased cell proliferation of TU212 and LCC cells (Fig. 6A-C). The opposite results were observed when overexpressing AQP9. Meanwhile, cell proliferation of TU212 and LCC cells was distinctly promoted by ZAP70 knockdown and was significantly decreased by its overexpression (Fig. 6D-F).

### AQP9 and ZAP70 up-regulation suppresses migration of laryngeal cancer cells

**Fig. 7:**
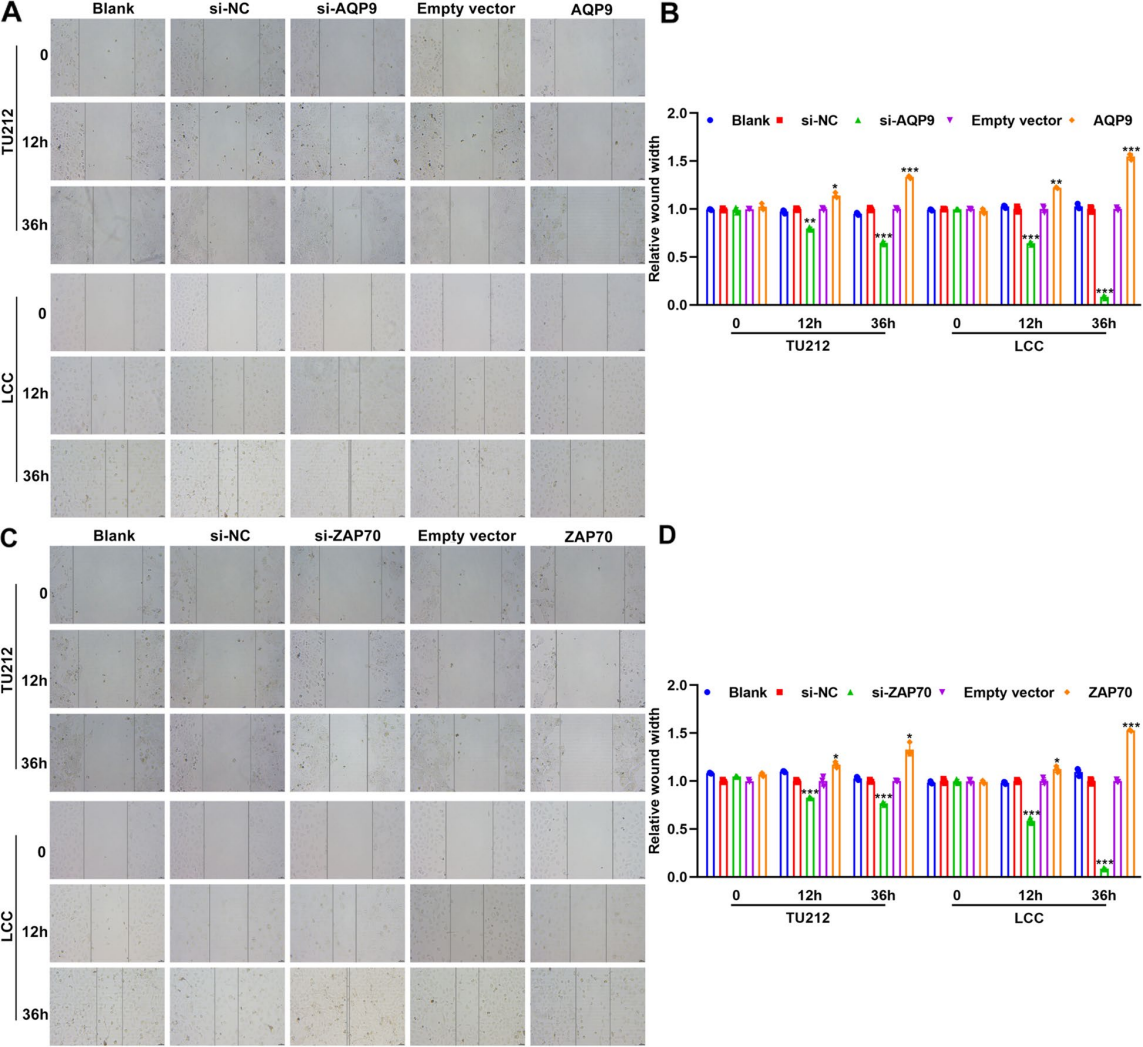
AQP9 and ZAP70 up-regulation suppresses migration of laryngeal cancer cells. **A**, **B** Wound healing for migration of TU212 and LCC cells after transfection with si-AQP9 or AQP9 overexpression plasmid. **C**, **D** Wound healing for migration of TU212 and LCC cells after transfection with si-ZAP70 or ZAP70 overexpression plasmid. *P < 0.05; **p < 0.01; ***p < 0.001

Wound healing assay was carried out to investigate the effects of AQP9 and ZAP70 on migration of laryngeal cancer cells. Our data showed that silencing AQP9 distinctly promoted migrated abilities of TU212 and LCC cells (Fig. 7A, B). Oppositely, migrated abilities were significantly suppressed by AQP9 overexpression. Furthermore, we also found that ZAP70 knockdown markedly facilitated migration of TU212 and LCC cells (Fig. 7C, D). The opposite results were observed when overexpressing ZAP70.

### AQP9 and ZAP70 up-regulation inhibits invasion of laryngeal cancer cells

**Fig. 8:**
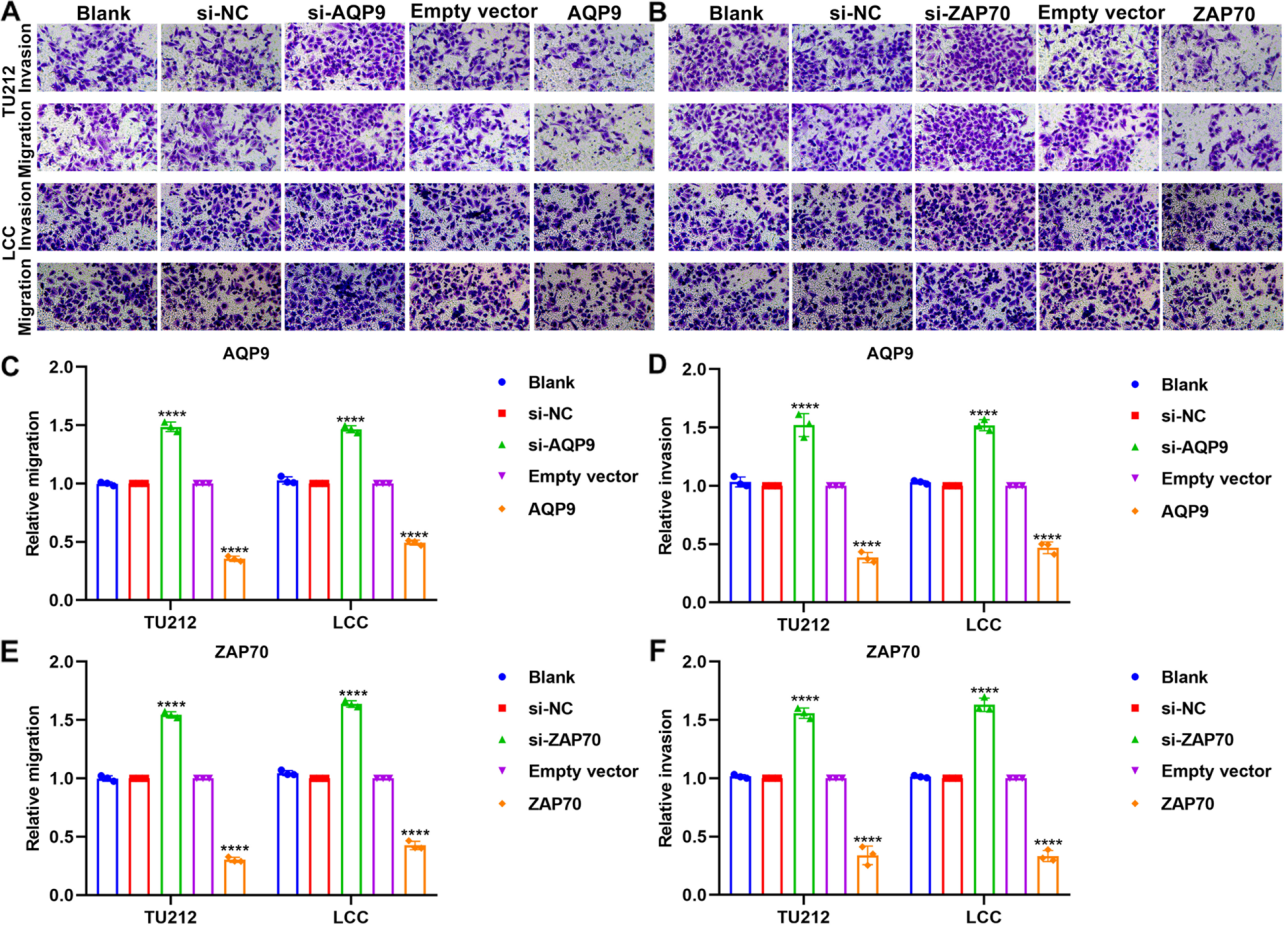
AQP9 and ZAP70 up-regulation inhibits invasion of laryngeal cancer cells. **A**, **B** Representative images of transwell assays. **C**, **D** Migration and invasion of TU212 and LCC cells under transfection with si-AQP9 or AQP9 overexpression plasmid. **E**, **F** Migration and invasion of TU212 and LCC cells under transfection with si-ZAP70 or ZAP70 overexpression plasmid. ****p < 0.0001

Transwell assays were applied for evaluating the roles of AQP9 and ZAP70 on migration and invasion of laryngeal cancer cells (Fig. 8A, B). As expected, AQP9 knockdown distinctly elevated migration and invasion of TU212 and LCC cells, while its overexpression markedly decreased migration and invasion of TU212 and LCC cells (Fig. 8C, D). Meanwhile, migrated and invasive capacities of TU212 and LCC cells were facilitated by ZAP70 knockdown (Fig. 8E, F). The opposite results were investigated under ZAP70 overexpression.

## Discussion

Laryngeal cancer represents the second most common head and neck cancer [23]. Its management is challenging and involves multidisciplinary care [24]. Despite much advance in treatment, overall survival has not prolonged partly due to alterations in therapeutic patterns and therapy-related toxicities [25]. IRGs have attracted attention as therapeutic targets. For example, Zeng et al. identified 23 co-expressed IRGs as immunotherapeutic biomarkers of laryngeal cancer that were distinctly associated with immune infiltrations and immune functions [26]. However, at present, there is still a lack of systematic research on IRGs in laryngeal cancer.

In this study, we firstly screened 315 abnormally expressed IRGs in laryngeal cancer compared to normal tissues in TCGA datasets by comprehensive analysis of DESeq2 and edgeR packages. By univariate cox regression analysis, correction of clinical factors and multivariate cox regression analysis, two IRGs AQP9 and ZAP70 were significantly correlated to survival outcomes of laryngeal cancer. Based on them, we characterized two molecular subtypes with distinct prognosis. To explore underlying mechanisms, we identified 24 differentially expressed IRGs between subtypes. These IRGs were significantly related to B cell activation, regulation of lymphocyte activation, positive regulation of lymphocyte activation, positive regulation of leukocyte activation, immunoglobulin mediated immune response, external side of plasma membrane, positive regulation of cell activation, B cell mediated immunity, regulation of leukocyte proliferation, receptor ligand activity, neutrophil degranulation and neutrophil activation involved in immune response as well as cytokine-cytokine receptor interaction, NF-kappa B signaling pathway, phagosome and primary immunodeficiency, demonstrating their critical roles in immune microenvironment. Furthermore, there were distinct differences in immune cell infiltrations between subtypes. This indicated that subtypes based on AQP9 and ZAP70 may reflect immune microenvironment of laryngeal cancer.

Dysregulation of gene expression may lead to tumor initiation and progression [27]. Our data showed that up-regulation of AQP9 and ZAP70 suppressed proliferation, migration and invasion of laryngeal cancer cells. The opposite results were investigated when their knockdown. Pan-cancer analyses identified that AQP9 was correlated with immune infiltration as well as acted as a prognostic factor in various cancers [16]. AQP9 up-regulation was detected in clear cell renal cell carcinoma specimens and was distinctly correlated to advanced clinicopathological factors and unfavorable survival outcomes [28]. In colorectal cancer, AQP9-mediated cell cycle arrest was correlated to RAS activation as well as improved chemosensitivity to 5-fluorouracil (5-FU) [29]. Colorectal cancer patients with high AQP9 expression exhibited favorable disease-free survival compared to those with its low expression. AQP9 expression was markedly down-regulated in hepatocellular carcinoma and was correlated to tumor size and number, TNM stage, five-year survival rate and metastases [30]. Also, it inhibited growth and metastases of hepatocellular carcinoma cells through Wnt/β-catenin pathway. Furthermore, it suppressed hepatocellular carcinoma stem cell properties through enhancing ROS/β-Catenin/FOXO3a pathway [31]. ZAP70 up-regulation enhanced chemokine-induced chronic lymphocytic leukemia cellular migration and arrest through valency regulating integrins [32]. Immunomodulatory drugs may activate NK cells through Zap-70 [33]. ZAP70 in B-cell lymphocytic leukemia cells correlated with the expression in NK cells represents a surrogate biomarker for mutation status [17]. Consistently, ZAP70 was in relation to laryngeal cancer prognosis [34]. ZAP70 mediated migration and invasion of prostate cancer cells [35]. Combining previous research, AQP9 and ZAP70 mediated laryngeal cancer progression.

Several limitations should be pointed out. Firstly, although our data demonstrated the roles of AQP9 and ZAP70 on proliferation, migration and invasion of laryngeal cancer cells, exact molecular mechanisms deserve in-depth observation. Secondly, despite the well predictive performance in prognosis, prospective research should be conducted for further assessing the predictive efficacy of AQP9 and ZAP70 in a larger cohort.

## Conclusion

Collectively, this study proposed two prognosis-related IRGs AQP9 and ZAP70. Two molecular subtypes based on AQP9 and ZAP70 may reflect immune microenvironment of laryngeal cancer. In vitro, their up-regulation inhibited proliferation, migration and invasion of laryngeal cancer cells. Our findings demonstrated that AQP9 and ZAP70 might be candidate therapeutic targets and prognostic signatures for laryngeal cancer, which may assist guide clinical therapy in the future.

## Supplementary Information

**Additional file 1: Supplementary Table 1**. Clinical information of laryngeal cancer patients.

**Additional file 2: Supplementary Table 2**. Differentially expressed IRGs in laryngeal cancer by DESeq2.

**Additional file 3: Supplementary Table 3**. Differentially expressed IRGs in laryngeal cancer by edgeR.

## Data Availability

All data generated or analysed during this study are included in this published article and its supplementary information files.

## Abbreviations

HTSeq: High-throughput sequencing
FPKM: Fragments Per Kilobase of transcript per Million mapped reads
TCGA: the Cancer Genome Atlas database
IRGs: Immune-related genes
CDF: Cumulative distribution function
GO: Gene Ontology
KEGG: Kyoto Encyclopedia of Genes and Genomes
RT-qPCR: Real-time quantitative polymerase-chain reaction
FBS: Fetal bovine serum
si-NC: siRNA negative control
CCK-8: Cell counting kit-8
EdU: 5-Ethynyl-2′-deoxyuridine
ROC: Receiver operator characteristic
AUCs: Area under the curve
